# Myocardial Adiponectin Isoform Shift in Dogs with Congestive Heart Failure—A Comparison to Hibernating Brown Bears (*Ursus arctos horribilis*)

**DOI:** 10.3390/vetsci4030035

**Published:** 2017-07-20

**Authors:** O. Lynne Nelson, Rachael M. Wood, Jens Häggström, Clarence Kvart, Charles T. Robbins

**Affiliations:** 1 Department of Veterinary Clinical Sciences, Washington State University, Pullman, WA 99164, USA; rwood@vetmed.wsu.edu; 2 Department of Clinical Sciences and Anatomy, Physiology and Biochemistry, Swedish University of Agricultural Sciences, Uppsala 750 07, Sweden; jens.haggstrom@slu.se; 3 Faculty of Veterinary Medicine and Animal Science, Swedish University of Agricultural Sciences, Uppsala 750 07, Sweden; clarence.kvart@slu.se; 4 School of the Environment and School of Biological Sciences, Washington State University, Pullman, WA 99164, USA; ctrobbins@wsu.edu

**Keywords:** low molecular weight adiponectin, high molecular weight adiponectin, chronic valvular heart disease, endocardiosis, hibernation

## Abstract

Adiponectin is the most abundant plasma adipokine, and is well known for its role in energy homeostasis and cardiac protection. In humans with dilated cardiomyopathy, myocardial adiponectin protein expression is reduced compared to normal hearts and has been implicated in the pathology of cardiomyopathy. Serum adiponectin levels are often conflicting, with higher levels associated with poor survival in humans with congestive heart failure (CHF). We evaluated adiponectin serum concentrations and myocardial protein expression in dogs with naturally occurring myxomatous mitral valve disease and CHF. We compared the findings to active and hibernating brown bears as bears are adapted to endure an extreme period of low cardiac output during their annual hibernation. Bears exhibited largely the active high-molecular weight (HMW) versus the low-molecular weight isoforms of myocardial adiponectin (HMW:LMW = 6.3) during both the active period and hibernation, while healthy dogs exhibited a more balanced mix of isoforms. Dogs with CHF expressed predominately HMW isoforms of adiponectin (HMW:LMW = 12.5), appearing more similar to bears. In contrast to humans, serum adiponectin was significantly lower in dogs with CHF and lowest levels in the severest CHF class. In both dogs and bears, myocardial adiponectin was expressed independent of circulating adiponectin concentrations, suggesting a local regulatory mechanism within the heart.

## 1. Introduction

Adiponectin is a primarily adipose tissue-derived cytokine that plays a key role in both metabolic and cardiac health. Adiponectin is the most abundant plasma adipokine, and is well known for its role in energy homeostasis and insulin sensitivity [1,2,3]. It is an essential protein for animals living in highly seasonal environments which must rely on annually switching from lipogenic to lipolytic states [4,5,6,7]. In contrast to other adipokines, serum adiponectin is inversely related to visceral obesity in humans [1,8]. Clinically, serum adiponectin concentrations are also inversely related to the risk of developing type II diabetes and cardiovascular disease, but directly related to decompensated congestive heart failure (CHF) [1,8,9,10,11,12,13]. Thus, these seemingly conflicting results are generating much interest into the systemic modulatory effects of this adipokine. 

Adiponectin regulates metabolism in part by promoting the phosphorylation and activation of AMP-activated protein kinase (AMPK) via the AdipoR1 receptor in skeletal muscle, adipose, and endothelial cells where it regulates glucose and lipid metabolism. The AdipoR2 receptor is expressed primarily in the liver, and its activation leads to increased insulin sensitivity [10,14,15,16]. The third adiponectin receptor—T-cadherin—is expressed predominantly in the heart and vasculature, and produces diverse myocardial and vascular protective effects, such as suppression of myocardial remodeling, and reduction of reactive oxygen species and pro-inflammatory cytokines [17,18]. There are multiple oligomeric forms of adiponectin that affect its activity. The isoforms are grouped into low-molecular weight (LMW, predominately in serum) and high-molecular weight (HMW, predominantly intracellular) [19,20]. Levels of the HMW isoform have better correlations with insulin sensitivity than total adiponectin, suggesting that the HMW isoform is the active form [21,22,23,24,25]. T-cadherin exclusively binds with HMW adiponectin. Studies in rodents deficient in T-cadherin have demonstrated pathologic cardiac hypertrophy, and worsening of inflammation and myocardial reperfusion injury [17]. Additional studies in animal models have demonstrated that increased expression of adiponectin can improve systolic function, inhibit protein synthesis, and retard cardiac remodeling [25,26,27,28]. In humans with dilated cardiomyopathy (DCM), myocardial adiponectin protein expression is reduced compared to normal hearts, and has been implicated in the pathology of cardiomyopathy [29]. Adiponectin is produced by the myocardium, and is released in proportion to the extent of left ventricular dysfunction which may in part explain the increased serum levels seen in humans with CHF [30]. 

Heart failure is no longer considered to be a single organ disease, but is now seen as a complex multisystem syndrome involving hemodynamic, neurohormonal, and metabolic alterations. Adiponectin has recently emerged as an important metabolic component [2,31]; however, interpretation of serum adiponectin concentration in human heart disease has been conflicting. On one hand, higher levels of serum adiponectin in the general population are considered healthy and are associated with reduced risk of diabetes mellitus, insulin resistance, systemic hypertension, and cardiovascular events [1,13,32,33,34]. On the other hand, low adiponectin levels are observed in heart disease without CHF, while the highest concentrations are seen in patients with CHF from any cause and are associated with poor survival. Along with the knowledge of adiponectin’s reputed beneficial effects on the heart, this U-shaped relationship of serum adiponectin in cardiac disease has been designated as “the adiponectin paradox” [34,35]. It is unclear if adiponectin has a negative impact on cardiac pathophysiology or if levels may rise to mitigate robust neurohormonal and metabolic impairment in CHF.

We were curious as to the roles of adiponectin in non-human species that manifest cardiac disease and CHF compared to species that might be considered to endure a natural hemodynamic “stress” of extremely low cardiac output. We compared naturally occurring canine CHF due to myxomatous mitral valve disease (MMVD) to hibernating brown bears (*Ursus arctos horribilis*). The reduction in cardiac output of these two conditions has been previously well documented. We felt it could be valuable to contrast native compensatory processes of disease manifestation to a natural physiologic process, as oftentimes the naturally adaptive response may shed light on the potential mechanisms underpinning the maladaptive response. Additionally, bears and dogs are close relatives in the Carnivora clade of mammals, and in general adiponectin appears to be well-conserved across species. We chose to compare canine MMVD (also known as endocardiosis or chronic valvular heart disease), as it is the most common acquired heart disease in dogs that presents for management of CHF. The prevalence of MMVD is high in older smaller breed dogs, with up to 85% showing some evidence of the disease at necropsy by 13 years of age [36,37]. The cause of MMVD is unknown, but the age of onset appears to have an inherited component in some dog breeds [38,39]. Bears are well known for their annual hibernation, where heart rate and cardiac output are reduced to 25% of the active season and maintained at this level for 4–6 months without feeding [40,41,42,43]. Hibernation is a natural physiological condition, and thus cardiovascular compensatory adaptations must occur for the myocardium to remain healthy and efficient during a long period of extremely low cardiac output. We hypothesized that active HMW myocardial adiponectin would increase in bears during hibernation if its presence is cardioprotective and associated with an altered myocardial workload. We hypothesized that myocardial HMW adiponectin would decrease and serum adiponectin would increase in dogs with CHF, reflecting decompensated CHF status similar to humans. Comparing a natural bradycardic state in bears that would create cardiac failure in other animals to a pathologic cardiac state in dogs may highlight adaptive processes. 

## 2. Materials and Methods 

### 2.1. Dogs

Healthy dogs (*n* = 18) and dogs with CHF (*n* = 18) were used to assess serum adiponectin. Dogs were included for sampling in the healthy group if the dog had no reported signs of systemic or cardiopulmonary illness and had a normal cardiovascular examination. The breeds of dogs included were: Beagle, Chihuahua (2), Maltese, Miniature Australian shepherd (2), Miniature Dachshund (2), Norfolk terrier, Pug, and eight mix-breeds. These dogs ranged in age from 4–12 years (mean weight: 8.7 kg). Dogs in the CHF group presented with clinical signs consistent with pulmonary edema and were ultimately diagnosed with decompensated MMVD by radiography and echocardiography. Dogs included for sampling in this study were classified into stage C or D (nine in each stage) according to the guidelines for the diagnosis and treatment of MMVD [36]. The breeds of CHF dogs included were: American Cocker Spaniel (2), Beagle, Brittany, Brussels Griffon (2), French Bulldog, Maltese (2), Miniature Schnauzer (2), Pembroke Welsh Corgi, Silky Terrier, Toy Poodle, and four mix-breeds. These dogs ranged in age from 9–14 years (mean weight: 9.4 kg). Blood samples were collected, spun, and stored at −80 °C. 

Canine left ventricular (LV) myocardium was collected from six normal dogs euthanized for reasons unrelated to this study and six dogs that died or were euthanized due to CHF caused by MMVD. The normal dogs ranged in age from 4–9 years and were all mixed-breed (mean weight: 14.3 kg). 

The normal dogs did not have an echocardiogram performed prior to death, but no gross cardiac abnormalities were noted on necropsy. The CHF dogs breeds were: Cavalier King Charles Spaniel (3), Miniature Dachshund, Toy Poodle, and terrier-mix. The CHF dogs ranged in age from 9–14 years (mean weight: 9.3 kg). All samples were snap frozen in liquid nitrogen within 30 min of death, and then stored at −80 °C until use. Dogs were client-owned, and owner consent was required for all sampling.

### 2.2. Bears

Sixteen brown bears (*Ursus arctos horribilis*) were used to assess serum adiponectin (4 males, 12 females). The age range was 2–22 years. All animals were housed at the Washington State University Bear Research, Education and Conservation Center. The animals were maintained according to the Bear Care and Colony Health Standard Operating Procedures approved by the Washington State Institutional Animal Care and Use Committee (Animal Subject Approval Form #3054) based on the U.S. National Institutes of Health guidelines. Hibernation began in early November, and feeding resumed the second week of March. Bears hibernated in pairs in unheated pens with continuous access through a small door to an outdoor area. The dens were monitored with surveillance cameras (Silent Witness, Surrey, BC, Canada) which confirmed that bears were recumbent for the hibernation period. Bears were anesthetized with tiletamine HCl/zolazepam HCl (5 mg/kg during the active phase and 2 mg/kg during hibernation) given intramuscularly. Due to the unique seasonal physiology of this species, blood samples were collected in serum tubes monthly throughout the year, spun and stored at −80 °C within 1 h. Body weights were recorded monthly from April to November in ten bears to correlate weight gain with circulating adiponectin levels in this species. Percent monthly weight gain was recorded to correct for the wide individual variation in size of bears. 

Left ventricular (LV) myocardium was collected from 12 grizzly bears (6 male, 6 female) euthanized for reasons unrelated to this project, generally the completion of ecology-related projects. The bears were considered healthy at the time of euthanasia. The age range was 3–22 years. Tissue collection was during active and hibernation periods (*n* = 6 for each group). The active period tissue collection was during the months of June, July, and August. The hibernation collection period was during the months of December and January. All samples were snap frozen in liquid nitrogen within 30 min of death, and then stored at −80 °C until use. All animal protocols were approved by Washington State University’s Institutional Animal Care and Use Committee (Animal Subject Approval Form #3054). 

### 2.3. Serum Adiponectin

Circulating adiponectin concentrations were quantified using a Mouse/Rat Adiponectin ELISA kit (B-Bridge International Inc., Mountain View, CA, USA) that has been previously validated in dogs and bears [44]. We chose to use one kit/method for both species versus using a canine ELISA kit which have been validated for dogs but not for bears. Additionally, we avoided using two different test kits, which could introduce assay variability. 

### 2.4. Western Blot Protocol

Left ventricular free wall myocardium samples were used to evaluate protein levels of adiponectin by Western Blot. Approximately 150 mg of tissue was frozen in liquid nitrogen and ground to a fine powder using a mortar and pestle. The tissue was transferred to a 7 mL tissue homogenizer and allowed to temper in a −20 °C freezer for 30 min, then homogenized in 1 mL Pierce IP lysis buffer with 10 μL Halt Protease Inhibitor cocktail (Thermo Fisher Scientific, Rockford, IL, USA). The tissue lysate was transferred to a 1.5 mL Eppendorf tube and centrifuged at 13,000 × *g* at 4 °C for 10 min. The supernatant was collected, and protein concentration determined by the bicinchoninic acid assay (BCA) method and stored at −80 °C until use. 

Velocity sedimentation was used to separate the adiponectin isomers as previously described [45]. Five-hundred micrograms of protein was diluted in 10 mM HEPES, pH 8, 125 mM NaCl, and layered on a 5–20% sucrose gradient in 10 mM HEPES, pH 8, 125 mM NaCl. The gradient was spun on an ultracentrifuge at 55,000 rpm for 4 h at 4 °C. Fractions were removed in 150 μL aliquots (labeled fractions 1–14), taken from the top of the gradient, and stored at −80 °C until use. 

**Figure 1:**
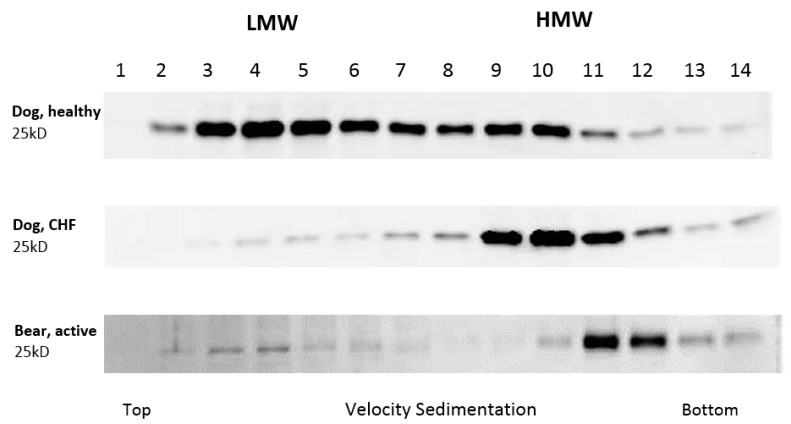
Identification of myocardial adiponectin fractions from velocity sucrose gradient sedimentation in a healthy dog, dog with congestive heart failure (CHF), and an active bear. Fractions 3–6 contain the low-molecular weight form (LMW) and 9–12 contain the high-molecular weight form (HMW).

Ten microliters of each fraction was added to 6× Laemmli buffer and heated to 95 °C for 5 min to denature the protein. Protein samples were loaded onto a precast gel (12% Tris-HCL ReadyGel, Bio-Rad Laboratories, Inc., Hercules, CA, USA) along with a protein standard (Precision Plus Protein Standards, Bio-Rad Laboratories, Inc., Hercules, CA, USA) and run at 150 V for one hour in 1× Tris/Glycine/SDS (TGS, Bio-Rad Laboratories, Inc., Hercules, CA, USA). The protein was then transferred to 0.2 μm pore nitrocellulose membrane in 20% methanol in 1× TGS transfer buffer for 3 h at 200 mA. The membrane was blocked in 5% nonfat dry milk in Tris Buffered Saline with 0.05% Tween 20 (TBS-T) at room temperature for one hour. The membrane was washed three times for 3 min with TBS-T and primary antibody 1:1000 rabbit anti-adiponectin (Sigma-Aldrich, St Louis, MO, USA) and 1:1000 anti-adipoR1 (Thermo Fisher Scientific, Rockford, IL, USA) diluted in 1% bovine serum albumin (BSA) in TBS-T was placed on the membrane and allowed to incubate overnight at 4 °C on a rocker. The membrane was washed five times in TBS-T for 5 min and then allowed to incubate in secondary antibody (1:20,000 Immun-Star Goat anti-rabbit HRP conjugate and 1:10,000 Precision Protein StrepTactin-HRP conjugate, Bio-Rad Laboratories, Inc., Hercules, CA, USA) diluted in TBS-T at room temperature for one hour on a shaker. A commercial kit (Immun-Star WesternC Chemiluminescent kit, Bio-Rad Laboratories, Inc., Hercules, CA, USA) was used according to manufacturer’s instructions for the detection of secondary antibody. Images were captured using a gel imaging system (ChemiDoc XRS Imager, Bio-Rad Laboratories, Inc., Hercules, CA, USA). Band density targeting adiponectin was determined for fractions obtained from sucrose gradients and plotted for each sample. Fractions 3–6 contained the LMW isoforms of adiponectin, and fractions 9–12 contained the HMW isoforms (Figure 1). 

### 2.5. Statistical Analysis

Analysis was performed using commercial statistical software (JMP SAS, Cary, NC, USA). For serum samples and Western blot samples, statistical analysis was performed using a decision tree. For paired data (measured on the same animal), the normality of the protein differences was assessed using the Shapiro–Wilk test. If significant at a level of 0.05, the nonparametric Wilcoxon signed rank test was used. If not significant, the paired *t*-test was used. Both tests were conducted to evaluate differences in protein between hibernating and active bears, and healthy dogs and dogs with CHF. Paired-samples *t*-tests have been shown to be appropriate with extremely small sample size, specifically when the within-pair Pearson coefficient is high [46]. For unpaired data, equal variances were assessed using Levene’s test, and normality of residuals were assessed using the Shapiro-Wilk test. If normality was achieved, then a two-sample *t*-test with either equal variances (Levene’s test not significant) or unequal variances (Levene’s test significant) was used. If normality was not achieved, the nonparametric Wilcoxon rank-sum test was used. A *p*-value of <0.05 was considered significant. All data are presented as mean ± standard deviation. 

## 3. Results

**Figure 2:**
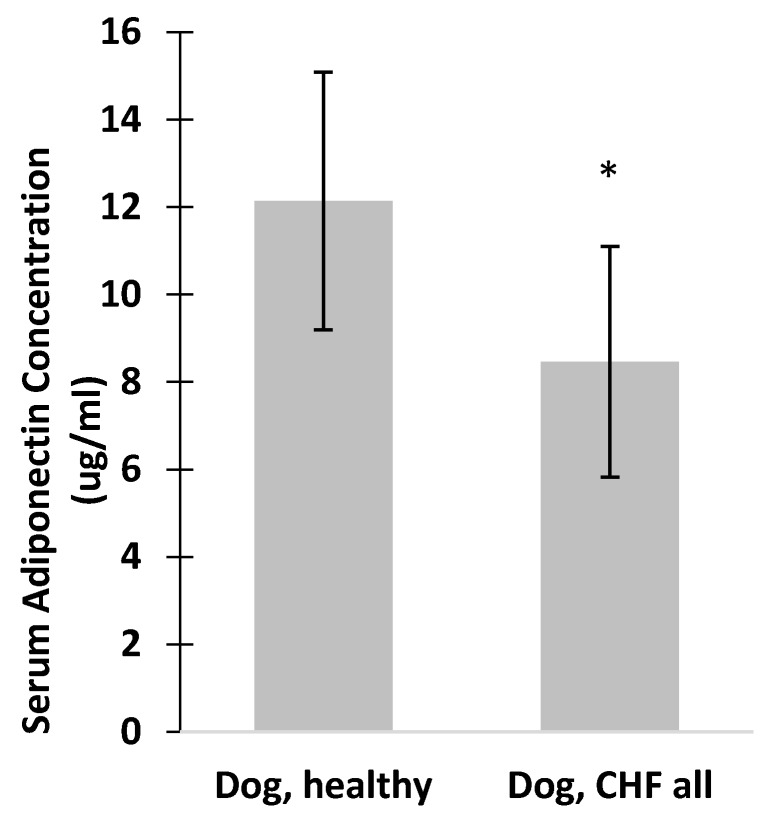
Mean serum adiponectin concentration was significantly higher in healthy dogs (*n* = 18) than in dogs with all classes of CHF (*n* = 18), * *p* = 0.001.

Mean serum adiponectin concentration was significantly higher in healthy dogs (12.1 ± 2.9 μg/mL, *n* = 18) than in dogs with all classes of CHF (8.4 ± 2.6 μg/mL, *n* = 18, *p* = 0.001; Figure 2).

**Figure 3:**
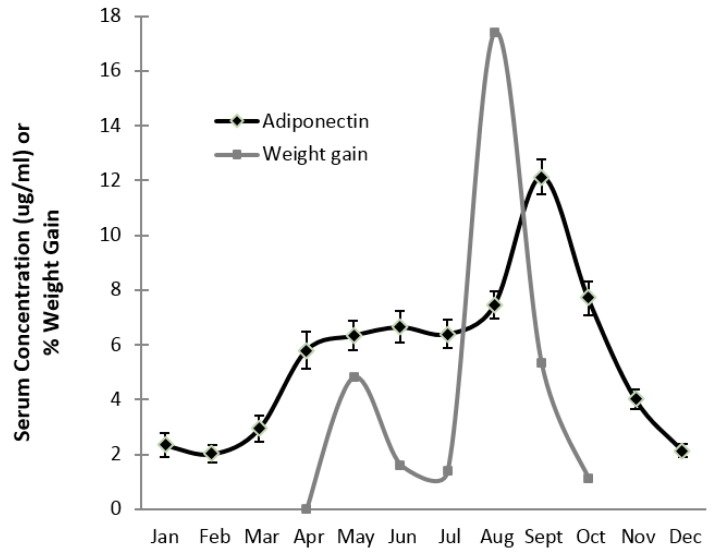
Monthly measurement of serum adiponectin compared to percent weight gain in 16 brown bears. Adiponectin data presented as means ± SD. The concentration of adiponectin mirrored weight gain through the active season (April–October) and peaked as rapid weight gain occurred in August and September. Serum adiponectin was significantly lower during hibernation (November–February) relative to the summer active period.

The serum concentration of adiponectin in bears varied seasonally, and was significantly lower during hibernation (November–February, 2.6 ± 0.8 μg/mL) than during the summer active period (April–August, 6.5 ± 0.5 μg/mL) (Figure 3). Its concentration increased dramatically in September (12.1 ± 1.5 μg/mL) after rapid weight gain occurred in August (fall hyperphagia). Adiponectin declined rapidly in October and November. 

**Figure 4:**
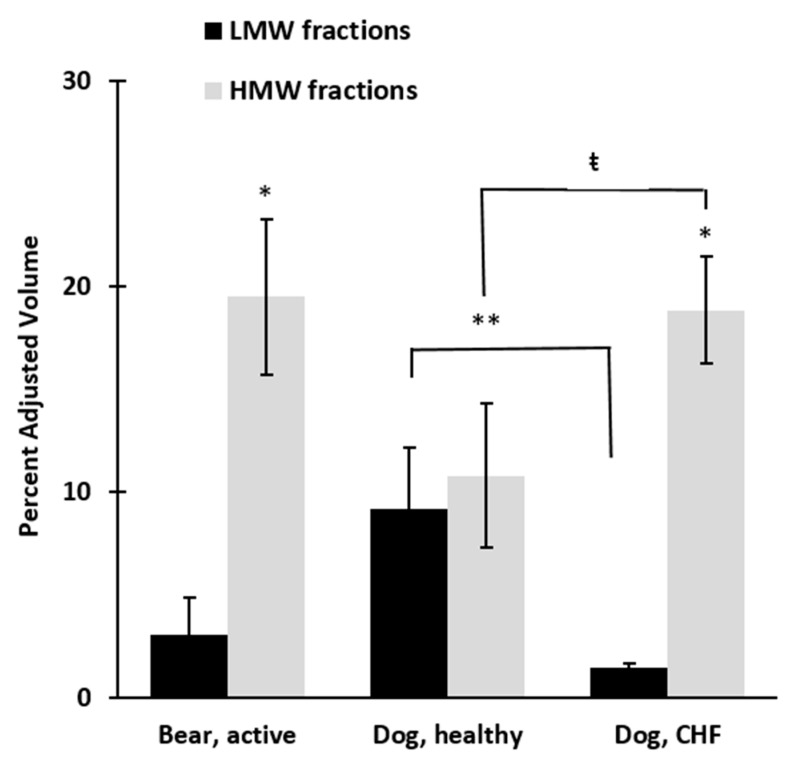
Pooled LMW and HMW fractions of myocardial adiponectin protein in six active bears, six healthy dogs, and six dogs with congestive heart failure (CHF). Fractions 3–6 contain the LMW isoforms of adiponectin and fractions 9–12 contain the HMW isoforms. Bears expressed 6.3 times greater concentration of HMW isoforms relative to LMW isoforms in both active period and hibernation. Healthy dogs expressed similar amounts of adiponectin isoforms, but dogs with CHF expressed 12 times greater concentrations of the HMW isoforms. * *p* < 0.0001; ** *p* = 0.005; ŧ *p* < 0.001.

**Figure 5:**
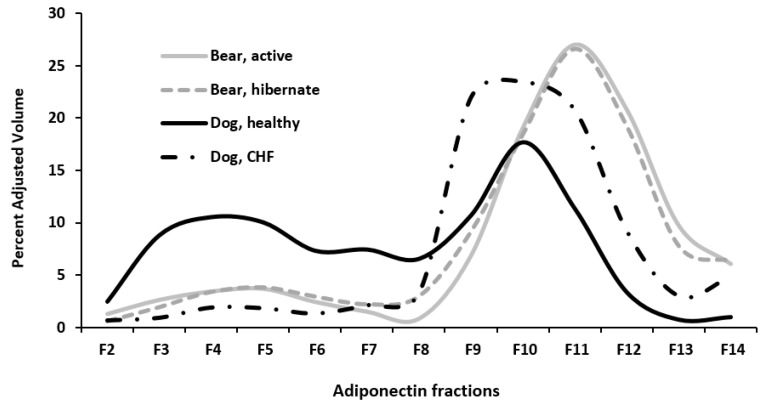
Band density targeting myocardial adiponectin was determined for fractions obtained from sucrose gradients and plotted for each group. Fractions 3–6 contain the LMW isoforms of adiponectin and fractions 9–12 contain the HMW isoforms. All bears expressed a higher concentration of HMW isoforms relative to LMW isoforms of adiponectin, and no significant difference was found between the active (*n* = 6) and hibernating bears (*n* = 6). Healthy dogs (*n* = 6) expressed similar myocardial concentrations of LMW and HMW adiponectin isoforms, whereas CHF dogs (*n* = 6) expressed significantly greater concentrations of HMW relative to LMW adiponectin.

Healthy dogs expressed similar myocardial concentrations of HMW (10.8 ± 3.4 μg/mL) and LMW (9.2 ± 2.9 μg/mL, *p* = 0.46) adiponectin isoforms, whereas CHF dogs expressed significantly greater concentrations of HMW isoforms (18.8 ± 2.6 μg/mL, *p* < 0.001) and significantly lower concentrations of LMW isoforms (1.5 ± 0.2 μg/mL, *p* = 0.005) adiponectin (Figure 4 and Figure 5). The ratio of HMW:LMW adiponectin expression in the healthy dog myocardium was 1.2, whereas the ratio of HMW:LMW adiponectin expression in dogs with CHF was 12.5. The increase in myocardial HMW adiponectin in CHF dogs was contrary to the decreasing circulating serum concentration in this group. Bears expressed a higher concentration of HMW isoforms in both the active: (19.5 ± 3.8 μg/mL) and hibernation states (18.9 ± 2.6 μg/mL) relative to LMW isoforms (active: 3.1 ± 1.8 μg/mL, hibernation: 3.0 ± 1.6 μg/mL, *p* ≤ 0.0001; Figure 4 and Figure 5), and the ratio of HMW:LMW isoforms did not change seasonally (*p* = 0.93). Cardiac adiponectin protein expression was independent of circulating adiponectin seasonal changes.

## 4. Discussion

The healthy dog myocardium expressed similar concentrations of HMW versus LMW adiponectin proteins, while CHF dogs expressed predominately the HMW isoforms (more than 12 times the LMW isoforms). The CHF dogs mirrored the bears’ myocardial adiponectin expression, as bears exhibited a predominance of HMW adiponectin in both the active and hibernating seasons (Figure 4 and Figure 5). High molecular weight adiponectin is the more active form, and could serve as a compensatory response for myocardial pathology in the dog. Animal model studies have demonstrated that increased expression of myocardial adiponectin can improve systolic function, inhibit protein synthesis, and retard cardiac remodeling [25,26,27,28]. As such, HMW adiponectin may be compensatory or protective in dogs with MMVD. In humans with DCM, myocardial adiponectin expression is reduced compared to normal individuals, independent of serum concentrations, implicating adiponectin in the pathology of the disease [29]. Since cardiomyocytes synthesize and secrete adiponectin, and blockage of protein secretion induces upregulation of adiponectin receptors, this suggests the existence of an auto/paracrine regulation within the heart [29,30]. It is unclear why myocardial adiponectin expression is increased dogs with CHF due to MMVD compared to the decrease seen in humans. The reason may be linked to a species-specific physiologic response or due to the different type of cardiac condition evaluated. Myxomatous mitral valve disease is a condition that causes volume overload and compensatory eccentric hypertrophy of otherwise normal cardiomyocytes, whereas DCM is a primary dysfunction of the cardiomyocyte [29,36]. A dysfunctional response may be more likely to result from primary muscle defect such as DCM. 

In addition, adiponectin expression in humans with cardiac disease may be confounded by high body mass index or type II diabetes, as there are more clear effects on adiponectin in humans with these conditions than in dogs [1,15,47,48]. High molecular weight adiponectin is also predominately expressed in the bear. Brown bears may need to maintain HMW adiponectin, as they have dramatically reduced cardiac output during hibernation, have evolved to accumulate large fat deposits in the fall, and subsequently switch from a lipogenic to lipolytic state as they enter hibernation [5]. In both the bear and dog, myocardial adiponectin is expressed independent of circulating adiponectin levels suggesting a local regulatory mechanism within the heart.

In contrast to humans, dogs with CHF had significantly reduced serum adiponectin concentrations (Figure 2). It is unknown if the discordance in serum level represents a species-related difference, a difference in cardiac disease evaluated, increased adiponectin utilization, or perhaps a decompensatory process. Discrepant studies suggest body condition score and neuter status of dogs may be important in interpreting serum adiponectin levels. These factors were not accounted for in this study. Total adiponectin is measured in serum, which consists of mainly the LMW isoform [1,19,20]. Total adiponectin could theoretically decline due to utilization of the HMW isoform and clearance from the serum, although this has not been evaluated in dogs or humans. 

Serum adiponectin in bears mirrored the change in weight gain over the summer months, then serum levels rapidly declined once hibernation began (Figure 3). The annual requirements for massive fat accumulation and maintenance of glucose metabolism to survive hibernation imply that adiponectin is directly related to weight gain and differs from the negative relationship that is found in humans. The association of adiponectin to percent body fat in active bears has been described [5]. Adiponectin is then uncoupled with adiposity in bears during hibernation, and marks the switch to insulin resistance, facilitating a lipolytic state [5]. In winter, bears show some similarity to humans, as very low levels of serum adiponectin are also used as a marker of insulin resistance in humans [1]. Due to the multiple metabolic functions of adiponectin (in heart, fat, vascular, liver, and muscle tissue), serum levels are not likely to be an adequate marker of tissue stores in the heart. We suspect myocardial adiponectin to be more directly related to cardiac physiology requirements and less affected by peripheral factors. For example, cardiac expression of adiponectin is maintained during extremely low cardiac output in hibernation (despite low circulating levels), implying that adiponectin is necessary for cardiac metabolism during this time. Thus, the upregulation of cardiac HMW adiponectin in the dog could suggest that adiponectin is also desirable in CHF due to MMVD. These data underscore the value of comparative physiology research by highlighting the value of contrasting native compensatory responses to disease manifestation. This approach could detect potential mechanisms of pathology or adaptation. 

### Study Limitations

The authors acknowledge that this is a small study with few numbers of animals, particularly of myocardial tissues. The diseases and states compared are somewhat different between species and patho/physiology observed. The proportion of measured serum adiponectin isoforms (LMH vs. HMW) is unknown, as only total serum adiponectin is measured.
